# Isolation and identification of three new chromones from the leaves of *Pimenta dioica* with cytotoxic, oestrogenic and anti-oestrogenic effects

**DOI:** 10.1080/13880209.2018.1448873

**Published:** 2018-03-22

**Authors:** Brian J. Doyle, Temitope O. Lawal, Tracie D. Locklear, Lorraina Hernandez, Alice L. Perez, Udeshi Patel, Shitalben Patel, Gail B. Mahady

**Affiliations:** a Department of Biology and Department of Biochemistry, Alma College, Alma, MI, USA;; b Department of Pharmacy Practice, University of Illinois at Chicago, Chicago, IL, USA;; c Department of Pharmaceutical Microbiology, University of Ibadan, Ibadan, Nigeria;; d Duke Clinical Research Institute, Duke University Medical Center, Durham, NC, USA;; e Centro de Investigaciones en Productos Naturales (CIPRONA), Natural Products Research Center, University of Costa Rica, San Jose, Costa Rica;; f Department of Pharmacy Practice, PAHO/WHO Collaborating Centre for Traditional Medicine, College of Pharmacy, University of Illinois at Chicago, Chicago, IL, USA

**Keywords:** Allspice, apoptosis, breast cancer, caspase, Costa Rica, ethnomedicine, oestrogen, Latin America, menopause

## Abstract

**Context:***Pimenta dioica* (L.) Merr. (Myrtaceae) is used in Costa Rican traditional medicine for women’s health. Our previous work showed that *P. dioica* extracts were oestrogenic.

**Objectives:** This work identifies phytochemicals from *P. dioica* that are responsible for the plant’s oestrogen-like activities.

**Materials and methods:***P. dioica* leaves were collected in Costa Rica in 2005. Fractions resulting from chromatographic separation of a methanol extract were tested at 50 μg/mL in a competitive oestrogen receptor-binding assay. Active compounds were isolated by HPLC and identified by NMR and MS. Pure compounds were tested at 1 μM in the oestrogen-responsive SEAP reporter gene assay. The effects on cell viability, cytotoxicity and apoptosis were investigated in breast cancer (MCF-7 and SK-BR3) and gastric cancer (AGS and NCI-N87) cell lines using the ApoTox-Glo and Caspase-Glo assays and qPCR.

**Results:** Quercitrin and three new chromones, including a 2-phenoxychromone, 6,8-di-C-methylcapillarisin (**1**) were isolated and identified. Compound **1** caused a 6.2-fold increase in SEAP expression at 1 μM (*p* < 0.05). This activity was blocked by the ER antagonist ICI 182,780. Compound **2** caused a 6.0-fold increase in SEAP, inhibited the growth of MCF-7, AGS and NCI-N87 cells (IC_50_ 54.27, 38.13 and 51.22 μg/mL, respectively), and induced apoptosis via caspase 8 and increased the Bax/Bcl-2 mRNA ratio in MCF-7 cells. Compound **3** was anti-oestrogenic in MCF-7 cells.

**Discussion and conclusions:** Compounds from *P. dioica* have oestrogenic, anti-oestrogenic and cytotoxic effects that may explain the ethnomedical use of this plant.

## Introduction

Menopause is defined as the termination of menstruation due to a reduction in the number and function of ovarian follicles, resulting in a decline in estradiol and progesterone production (Gold 2011). The menopausal transition (perimenopause) is a complex physiological process beginning at the early menstrual transition stage, when menstrual cycle length becomes irregular, and ending 12 months of amenorrhea (Nelson 2008; Harlow et al. 2012). During the menopausal transition, declining oestrogen and progesterone levels cause most women to experience vasomotor symptoms such as hot flashes and night sweats, as well as other symptoms such as anxiety, depression, mood swings, sleep disorders, vaginal dryness and joint pain (Nelson 2008).

In Costa Rica, women enter menopause around the same age as their American counterparts (∼50 years), and suffer from similar menopausal symptoms (Haines et al. 2005; Locklear et al. 2007, 2017; Palacios et al. 2010). Although hormone therapy is the gold standard for the management of menopausal symptoms, Latin American women use more natural therapies to manage their menopausal symptoms, including a wide range of herbal medicines to improve the quality of life in the peri- and postmenopausal periods (Michel et al. 2006; Locklear et al. 2007, 2017; Doyle et al. 2009).

As part of our work in Central America, we have identified more than 17 plant species used in Costa Rica to treat women’s reproductive health disorders with a focus on menopause (Locklear et al. 2007, 2017; Doyle et al. 2009). *Pimenta dioica* (L.) Merr. (Myrtaceae), (allspice leaves) was selected for further investigation based on its activity in preliminary screens for oestrogen-like effects (Doyle et al. 2009). *Pimenta dioica* is sold in Costa Rica as an herbal therapy for menopausal symptoms and is usually prepared as a decoction, infusion or as a tincture, alone or in combination with other herbs (Doyle et al. 2009). Preparations of *P. dioica* are further used in Costa Rica for the treatment of dysmenorrhea and dyspepsia, and extracts have also been shown to have antitumor effects (Zhang and Lockeschwar 2012). In addition, Cha et al. (2006) showed that extracts of allspice inhibit the growth of *Helicobacter pylori*, the bacteria responsible for dyspepsia and gastric cancer.

In our previous work, we found that extracts of the leaves of this plant bound to both the ERα and ERβ isoforms of the oestrogen receptor with preference for ERβ, as well as induced transcription of an oestrogen responsive luciferase gene in stably transfected MCF-7 cells (Doyle et al. 2009). In addition, the extracts also down-regulated the expression of PTGES in MCF-7 cells suggesting potential chemopreventive activities (Doyle et al. 2009). The present investigation aimed to isolate and identify the chemical constituents from a *P. dioica* leaf extract with oestrogen-like effects, and to evaluate their activities in ER binding, oestrogen-responsive reporter gene and in cancer cell assays. Bioassay-guided fractionation of the crude *P. dioica* extract was performed based on activity in the ERβ-binding assay. This resulted in the isolation of the known compound quercitrin, a new 2-phenoxychromone, 6,8-di-C-methylcapillarisin (**1)**, and two new glycosylated methyl chromones (**2** and **3**).

## Materials and methods

### Memorandum of agreement

This work was performed as a collaborative project between the University of Illinois at Chicago (UIC) and the University of Costa Rica (UCR) based on a Memorandum of Agreement signed by authorities from UIC and UCR.

### Plant collection and extraction

The leaves of *P. dioica* were collected in 2005 at Finca La Isla in Playa Negra, Limon Province, Costa Rica, and extracts were prepared at the Center for Natural Products Research (CIPRONA) at the UCR. Leaves were dried in an oven at 37 °C and ground in a hammer-mill to a course particle size. The plant material (1 kg dry weight) was then extracted by maceration in 5 L methanol twice overnight. The extract was filtered and partially dried *in vacuo* followed by lyophilization. Herbarium specimens were identified by Jorge Laurito-Gomez at the UCR and deposited in the herbarium at UCR (voucher #BD101).

### Cell culture and maintenance

Human gastric cancer cells, AGS and NCI-N87 were purchased from ATCC (Manassas, VA). Human breast adenocarcinoma cells, MCF-7 were a kind gift from Dr. Hyun-Young Jeong of the Department of Pharmacy Practice, UIC. It was grown and maintained in minimum essential medium Eagle with Earle’s salt and l-glutamine (MEM 1X; Corning Cellgrow, Manassas, VA). AGS (CRL-1739) was obtained from ATCC, grown and maintained in Kaighn’s modification of Ham’s F-12 with l-glutamine (ATCC). NCI-N87 obtained from ATCC was grown and maintained in RPMI 1640 medium (Gibco, Life Technology, Grand Island, NY). All growth media were supplemented with 10% FBS (Gibco, Life Technology, Grand Island, NY) and 1% penicillin/streptomycin (Gibco, Life Technology, Grand Island, NY). The cells were incubated at 37 °C in a humidified atmosphere of 5% CO_2_ and 95% air. At 80% confluency, the cells were harvested by adding 0.25% trypsin/EDTA and counted by means of trypan blue and haemocytometer. These cells were then re-suspended at appropriate concentration and plated for cellular assays.

### Cell viability assay

MCF-7 and AGS cells were seeded at 2.5 × 10^4^ cells in 100 µL/well while NCI-N87 was seeded at 5.0 × 10^4^ cells in 100 µL/well in opaque-walled 96-well plate. Control wells containing medium (supplemented with 10% FBS and 1% penicillin/streptomycin) without cells to determine background luminescence were also prepared. The cells were left to attach overnight in the plate. Culture medium was aspirated and fresh medium added to the wells before reconstituted extracts of *P. dioica* (methanol extract, 50% methanol fraction) and isolated compounds (**1**–**3**, and quercitrin) at 100, 50, 20, 10 and 5 µg/mL were added to experimental wells. Drug controls (5-fluorouracil and doxorubicin) and vehicle control (0.02% DMSO) wells were also prepared simultaneously, and the plate was incubated in a humidified incubator at 37 °C in an atmosphere of 5% CO_2_ for 72 h. At the end of the incubation period, the plate and its contents were equilibrated to room temperature for approximately 30 min. A 100 µL volume of CellTiter-Glo 2.0 Reagent (Promega Corporation, Madison, WI) was added to each well. The contents were mixed for 2 min on an orbital shaker to induce cell lysis and the plate was incubated at room temperature for 10 min to stabilize the luminescent signal. Luminescence signal was read using the Synergy HT Plate reader (Biotek, Winooski, VT) and Gen5 1.11 software. The IC_50_ defined as the concentration that causes 50% inhibition of cell growth after exposure to extract for 72 h was calculated using log (inhibitor) vs. normalized response analysis with GraphPad Prism 7 (GraphPad Software, Inc., La Jolla, CA).

### Apoptosis assay via ApoToxGlo™ triplex assay

Ninety-six (96) well assay plates containing MCF-7 cells in medium at a density of 1.0 × 10^4^ cells in 100 µL/well in triplicate were set up. After overnight incubation to allow attachment of cells, **2** at its IC_50_ concentration of 55 µg/mL and vehicle controls were added to the appropriate wells at a final volume of 100 μL per well. Plates were incubated appropriately before addition of 20 μL of viability/cytotoxicity reagent containing both GF-AFC substrate and bis-AAF-R110 substrate to all wells and briefly mixed by orbital shaking (300–500 rpm for ∼30 s). Plates were incubated for 30 min at 37 °C and fluorescence was measured at 400_ex_/505_em_ (viability) and 485_ex_/520_em_ (cytotoxicity). A 100 μL volume of Caspase-Glo^®^ 3/7 reagent was added to all wells, briefly mixed by orbital shaking (300–500 rpm for ∼30 s), and incubated for 30 min at room temperature. Luminescence was measured using the Synergy HT Plate reader (Biotek, Winooski, VT) and Gen 5 1.11 software to detect caspase activation.

### Apoptosis assay via Caspase-Glo^®^ 8

MCF-7 cells were seeded in triplicate at a density of 3.0 × 10^4^ cells in 100 µL/well in opaque-walled 96-well plates. The cells were left to attach overnight in the plate. Compound **2** at 55 µg/mL was added to the test wells. Caspase-Glo^®^ 8 Reagents (Promega Corporation, Madison, WI) were prepared according to manufacturer’s instruction and allowed to equilibrate to room temperature. The test plates containing treated cells as well as controls were removed from the incubator after 2 h of incubation and allowed to equilibrate to room temperature. A 100 μL volume of Caspase-Glo^®^ 8 Reagent was added to each well of a 96-well plate containing 100 μL of cell culture medium (blank), non-treated cells (negative control) in culture medium, and treated cells in culture medium. The contents of wells were gently mixed using a plate shaker at 300–500 rpm for 30 s and incubated at room temperature for 1 h. The luminescence of each sample was read using the Synergy HT Plate reader (Biotek, Winooski, VT) and Gen5 1.11 software.

### RNA isolation

MCF-7 cells were seeded at a density of 1 × 10^6^ cells per well in a six-well plate and incubated overnight for cell attachment. Total RNA was extracted from cells that had been treated with **2** at 55 µg/mL after 2 h incubation using Trizol (ThermoFisher Scientific, Waltham, MA) according to the manufacturer’s instructions. Vehicle-treated control cells served as the negative control. The concentration and quality of RNA were determined by measuring absorbance at 260 nm and 280 nm on SynergyHT Take 3 session (Biotek, Winooski, VT) and Gen5 1.11 software.

### Quantitative polymerase chain reaction

Total RNA was reverse transcribed and amplified using Power SYBR Green RNA-to-C_T_ 1-step kit (Applied Biosystems, Foster City, CA) as described by manufacturer using a Step-One Plus Real Time PCR System (Applied Biosystem, Foster City, CA). Primer sequences were selected from previously published papers and shown in Table 1. Briefly, each reaction was performed in triplicate in a 10 μL volume containing Power SYBR Green RT-PCR Mix (2×), 200 nM of each primers, RT Enzyme Mix (125×) and 100 ng RNA. The cycling conditions were as follows: 48 °C for 30 min, 95 °C for 10 min, followed by 50 cycles of 95 °C for 15 s and 60 °C for 1 min. PCR reaction specificity was confirmed by melt curve analysis at 95 °C for 15 s, 60 °C for 15 s, 95 °C for 15 s. The quantitation of gene expression was performed using β-actin as an endogenous control and relative to the calibrator (control cells) using the ΔΔCt method. Melting curves for each primer set were performed prior to the use of the primers.

**Table 1.** Sequences of primers used in real-time PCR analysis.

Gene	Forward primer sequence (5′–3′)	Reverse primer sequence (5′–3′)
β-Actin	TGACGTGGACATCCGCAAAG	CTGGAAGGTGGACAGCGAGG
Bcl-2	CGCATCAGGAAGGCTAGAGT	AGCTTCCAGACATTCGGAGA
Bax	TGCCAGCAAACTGGTGCTCA	GCACTCCCGCCACAAAGATG
p53	AAGTCTGTGACTTGCACGTACTCC	GTCATGTGCTGTGACTGCTTGRTAG
HER2	AACTGCACCCACTCCTGTGT	TGATGAGATCCCAAAGACC

### Oestrogen binding assay

A competitive radioligand-binding assay using full-length ERβ was used in the bioassay-guided fractionation of the crude methanol extract. The assay was performed as previously described (Obourn et al. 1993; Doyle et al. 2009). Briefly, recombinant human oestrogen receptor from insect Sf9 cells (α or β) was incubated with the test sample (50 μg/mL) plus 0.5 nM 3H-estradiol at 4 °C overnight. At the completion of incubation, 100 μL of a 50% hydroxylapatite slurry (in 40 nM Tris, pH 7.4, 1 mM EDTA, 1 mM EGTA) was added and allowed to bind the ER-ligand complex for 40 min. The hydroxylapatite was washed three times with 0.5 mL of 40 mM Tris, pH 7.4, 1 mM EDTA, 1 mM EGTA and 11 mM KCl. The hydroxylapatite pellets were suspended in 1 mL of ethanol and counted in 5 mL of scintillation fluid, and the receptor-bound 3H-estradiol was measured. At each step in the fractionation process, fractions were assayed, and the most active fraction was pursued for further fractionation. The median inhibitory concentration of **1** was determined by testing at concentrations of 20–100 μg/mL. All tests were performed in triplicate, and the results were determined from three independent experiments.

### Compound isolation

**Scheme 1.**
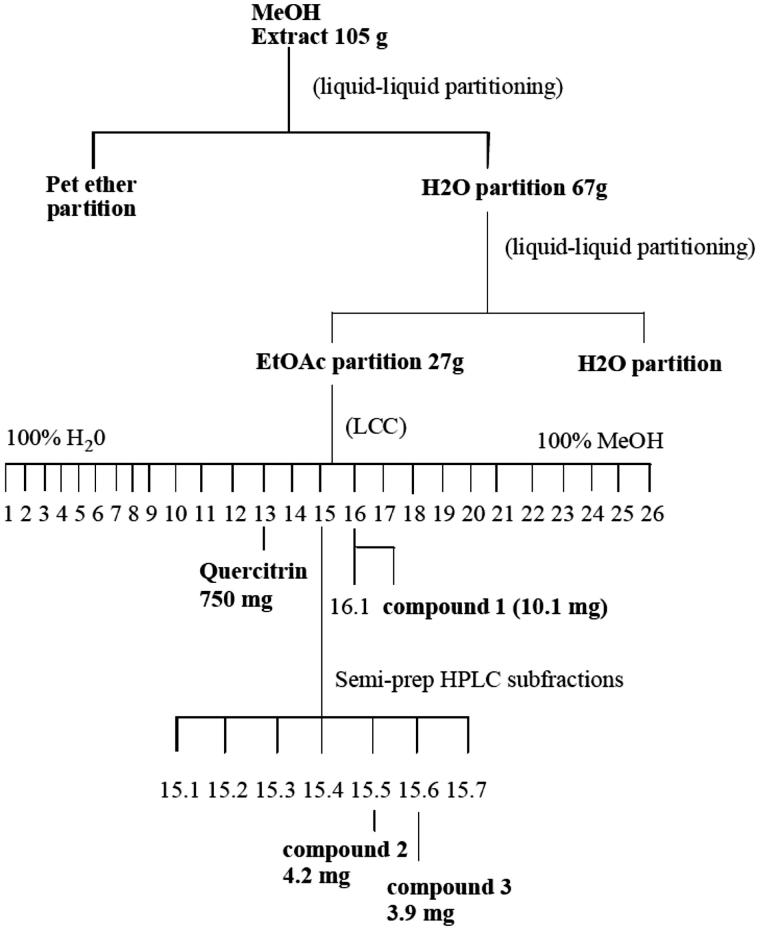
Flowchart for the isolation and identification of quercitrin, and compounds **1–3** from a methanol extract of the leaves of *P. dioica*.

The extract was first defatted by liquid–liquid partitioning in petroleum ether/water, and the aqueous extract was subsequently partitioned into EtOAc and aqueous portions (Scheme 1). The EtOAc portion bound to the oestrogen receptor while the aqueous portion did not. The EtOAc portion (27 g) was further separated by low-pressure column chromatography using C18 reversed phase silica gel in a 2 L column and eluted with a water:methanol solvent gradient ranging from 100% water to 100% methanol. Fractions were collected and then analysed on C18 silica gel thin layer chromatography plates developed with a mobile phase of water and methanol at a ratio similar to that with which the fractions eluted from the LC column. Fluorescence under UV light was used as the detection method. Fractions that were similar in appearance based on the TLC chromatogram were combined. The resulting fractions were then assayed for oestrogenicity in the oestrogen receptor competitive binding assay and for cytotoxicity in MCF-7 and SK-BR3 breast cancer cells or AGS and NCI-N87 gastric cancer cells. Active fractions were analysed on a Dionex Summit HPLC System (Thermo-Fisher Scientific, Waltham, MA) with photodiode array detector using a Waters Atlantis dC18 reverse phase analytical HPLC column (5 μm, 4.6 × 250 mm) (Milford, MA). Chromelan software Vs 6.5 was used for the data analysis. An isocratic solvent system was used with a methanol:water ratio similar to that with which each fraction eluted from the LC column. Further rounds of subfractionation were performed as necessary by semi-preparative HPLC. Fractions were dissolved in a minimal amount of methanol (∼100 mg/mL) and run on a Waters Atlantis dC18 semi-preparative column (10 μm, 10 × 150 mm). Injection volumes were 100–150 μL and flow rate was 3–6 mL/min. Samples were run under isocratic conditions using a solvent system consisting of between 30 and 60% MeOH. Sub-fractions were collected and analysed on an analytical HPLC column as described above.

### Structure elucidation of 1–3

The structures of **1**–**3** were elucidated using nuclear magnetic resonance (NMR) and mass spectroscopy (MS). NMR analysis was performed on a Bruker DRX-400 400 MHz spectrometer (Billerica, MA) using DMSO-*d*_6_ as the solvent. Spectra were processed and analysed using Xwin NMR software. Both ^1^H and ^13^C 1D experiments were performed, and 2D experiments, such as ^1^H–^1^H COSY, HSQC and HMBC, were performed as necessary. The molecular weights were obtained by MS using a Shimadzu LCMSITTOF spectrometer (Kyoto, Japan) with MeOH as the solvent. Samples were analysed in both positive and negative polarity modes using the ESI method of ionization.

### Determination of oestrogen agonist/antagonist effects

In addition to the ERβ binding assay, oestrogenic activity was determined by an oestrogen-responsive reporter gene assay consisting of MCF-7 cells transfected with a construct containing a secreted alkaline phosphatase (SEAP) gene that is induced through an oestrogen response element (ERE). MCF-7 (HTB-22) human breast cancer cells (obtained from American Type Culture Collection, Manassas, VA) were maintained in Dulbecco’s modified Eagle’s medium containing 10% FBS and 100 U/mL penicillin and 100 μg/mL streptomycin in 75 cm^2^ flasks. Forty-eight hours prior to transfection, the cells were maintained in phenol red free DMEM medium supplemented with 0.5% FCS, in a humidified atmosphere with 5% CO_2_ at 37 °C. For transfection, 0.5 × 10^5^ cells were seeded per well of a 96-well plate in OPTI-MEM supplemented with dextran-charcoal stripped FBS. After 4 h, serum concentration was reduced to 1%. Prior to transfection, cells were washed with DMEM and incubated in phenol red-free OPTI-MEM without FBS as described by Nozawa et al. (2005).

The cells were transfected with the reporter vector pERE-TA-SEAP, coding for the SEAP gene (Clontech, Palo Alto, CA) (Cullen and Malim 1992; Treeck et al. 2003). MCF-7 cells were transfected using 0.5 μL Lipofectamine 2000 (Invitrogen, Carlsbad, CA), and 0.1 μg plasmid DNA including the SEAP vector per well of a 96-well plate according to the manufacturer’s instructions. As a negative control, cells were transfected with reporter vector pTA-SEAP2 using Lipofectamine 2000. After 12 h of transfection, the media were replaced with phenol-free Dulbecco’s modified Eagle’s medium containing 10% dextran-coated charcoal stripped FBS. Cells were treated with 1 μM **1**–**3** with and without 10 nM E2 or 1 μM ICI 182,780. Estradiol was used as the positive control and DMSO as the negative control. Cells were treated for 12 h, then the medium was harvested, centrifuged and 25 μL from each well was transferred to a new 96 well plate. The SEAP activity was measured using the Great EscAPe SEAP Chemiluminescence High-Throughput Detection Kit (Clontech, Palo Alto, CA) using a 96-well luminometer according to the manufacturer’s instructions. To assess novel mechanisms of action other than ER, we tested the extract and fractions in a HER2/neu tyrosine kinase assays (Barbacci et al. 2003; HTScan HER2/ErbB2 kinase Assay Kit, Cell Signaling Tech., 96-well plates, high throughput), using the methods as described by the manufacturer.

### Statistical analysis

Statistical analysis was performed using one-way ANOVA followed by Tukey’s multiple comparison test as the *post hoc* analysis (GraphPad Software 7.0, La Jolla, CA). Statistical significance was ascribed to the data when *p* < 0.05.

## Results

### Structure elucidation of isolated compounds

The compounds were isolated using liquid–liquid partition chromatography, column chromatography and semi-preparative HPLC (Scheme 1).

Quercitrin (750 mg) was isolated as a yellow, amorphous powder and was identified by comparison of ^1^H and ^13^C NMR and high resolution MS data with previously published data for this compound (Ribeiro et al. 2002).

**Figure 1.**
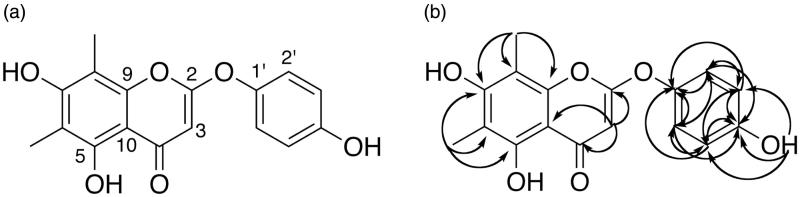
(a) Structure of compound **1**. (b) HMBC correlations (400 MHz, DMSO-*d*_6_).

Compound **1** (10.1 mg) was isolated as a white, amorphous powder. The molecular formula C_17_H_14_O_6_ was determined by high resolution MS ([M + H]^+^=315.0858 *m*/*z*), and the structure was determined to be 2-(*p*-hydroxyphenoxy)-6,8-dimethyl-5,7-dihydroxychromone (Figure 1) based on the results of 1D and 2D NMR experiments. *δ*^1^H (ppm): 13.0 (1H, s, 5-OH), 9.77 (1H, s, 4′-OH), 9.72 (1H, s, 7-OH), 7.20 (2H, d, *J* = 8.83, H-3′/5′), 6.86 (2H, d, *J* = 8.84, H-2′/6′), 5.09 (1H, s, H-3), 2.08 (3 H, s, 8-Me), 2.02 (3H, s, 6-Me). *δ*^13^C (ppm): 183.6 (C-4), 167.6 (C-2), 159.4 (C-7), 156.2 (C-4′), 150.1 (C-9), 143.1 (C-1′), 122.0 (C-2′/6′), 116.5 (C-3′/5′), 107.5 (C-6), 101.9 (C-8/10), 86.7 (C-3), 8.0 (6-Me/8-Me).

The substitution pattern in the A ring was determined based on HMBC correlations (Figure 1(b)) between both methyl groups and C-7 (*δ* C 159.4 ppm), suggesting that a hydroxy-substituted carbon, C-7, was positioned between the two methylated carbons, C-6 and C-8. The methyl group assigned to C-6 also correlated with C-5, which was assigned based on HMBC correlation with the hydroxyl proton at *δ* 13.0 ppm. Furthermore, NMR data for this compound are consistent with previously published NMR data for the 6,8-dimethyl-5,7-dihydroxyflavone syzalterin (Youssef et al. 1998). Also important was the observation that the upshifted carbonyl resonance was indicative of a flavone (double bond between C-2 and C-3), but the downshifted resonances of C-2 and C-1′ did not correspond to a simple flavone structure. Furthermore, the molecular formula derived from HRMS data suggested a sixth oxygen atom. HMBC and HSQC correlations clearly indicated a proton at C-3 rather than the –OH substitution typical of flavonols. Positioning the oxygen atom between C-2 and C-1′ creating a 2-phenoxy moiety results in the observed downshift of C-2 and C-1′ resonances. This is supported by previously published NMR data for the 2-phenoxychromone piliostigmin (Ibewuike et al. 1996).

**Figure 2.**
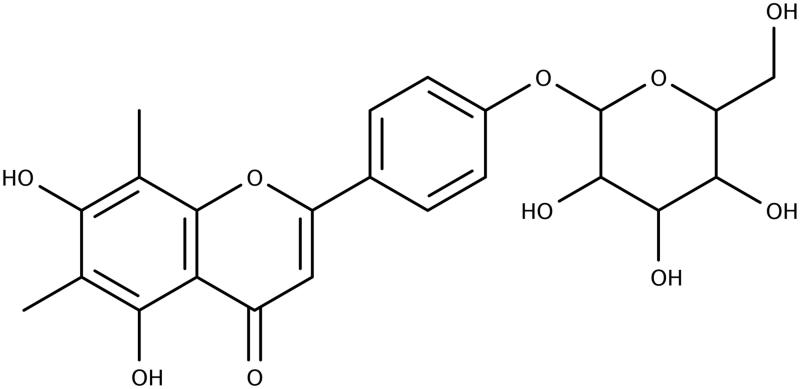
Structure of compound **2**.

Compound **2** was isolated as a yellow powder (4.2 mg) from fraction 15, sub-fraction 5 (15.5). Based on the data from 1D and 2D NMR, MS analysis, comparison with data from **1**, and study of the literature, **2** appears to be a new *O*-glycoside of syzalterin (6,8-dimethyl-5,7,4′-trihydroxyflavone) (Figure 2). The aglycone was identified as syzalterin through comparison of the NMR, MS and UV data with that previously published for the compound (Youssef et al. 1998). It is evident from the NMR data that a hexose, likely glucose, is present, and a NOESY correlation between the anomeric proton of the sugar and H-3′ is suggestive of linkage at position 4′.

MS analysis detected [M + H]^+^ at 461.1 *m*/*z* which is consistent with the molecular formula for syzalterin-4′-*O*-glucopyranose C_23_H_24_O_10_. *δ*^1^H (400 MHz, DMSO) 13.07 (1H, s, 5-OH), 8.04 (2H, d, *J* = 9.03, H-2′/6′), 7.21 (2H, d, *J* = 8.75, H-3′/5′), 6.89 (1H, s, H-3), 5.61 (1H, s), 5.39 (1H, s), 5.12 (1H, s), 5.06 (1H, s), 5.02 (1H, d, *J* = 6.66, H-1″), 4.6 (1H, s), 4.36 (1H, s), 3.70 (m), 3.5–3.1 (m), 2.30 (s, 8-CH_3_), 2.05 (s, 6-CH_3_). *δ*^13^C (400 MHz, DMSO) 182.2 (C-4), 162.7 (C-2), 160.3 (C-7/4′), 156.1 (C-5), 152.6 (C-9), 128.1 (C-2′/6′), 124.4 (C-1′), 116.7 (C-3′/5′), 107.2 (C-6), 103.6 (C-3/10), 102.0 (C-8), 100.0 (C-1″), 77.2, 76.6, 73.2, 69.7, 60.7, 8.4 (8-CH_3_), 8.1 (6-CH3).

**Figure 3.**
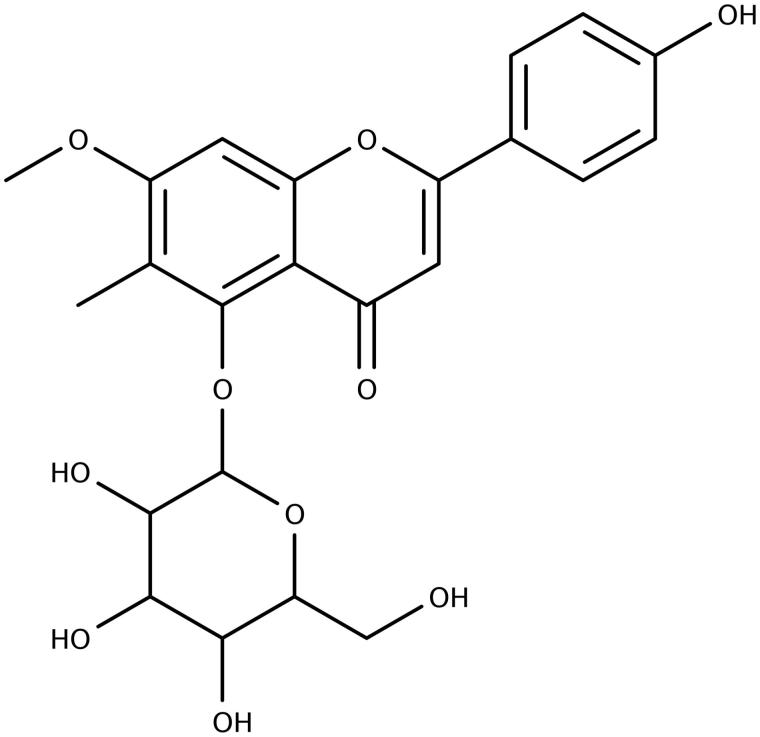
Structure of compound **3**.

Compound **3** was isolated as a white powder (3.9 mg) from fraction 15, sub-fraction 6 (15.6). Based on comparison of 1D and 2D NMR and MS data with that of **2**, **3** is the 5-*O*-glycoside of 7-methoxy-6-methyl-3,5,4′-trihydroxyflavone (Figure 3). The molecular weight of 476.1 amu was determined by MS and is consistent with the hypothesized structure. Compared to **2**, **3** is 16 amu heavier, corresponding to an extra oxygen atom in the structure. This oxygen is observed as a hydroxyl function in the ^1^H NMR spectrum visible as a broad singlet at 10.08 ppm. The signal due to the proton at C-3 in **2** is not observed, thus this hydroxyl group was assigned to this position making **3** a flavonol. The hydroxyl group in this position also accounts for the upshifted C-4 carbonyl resonance at 176 ppm and the upshifted C-2 resonance at 144 ppm compared to 182.2 and 162.7, respectively, for **3**. A singlet peak is observed, however, due to a proton that has been assigned to C-8 based on HMBC correlations with the methylated carbon at C-6 (107.5 ppm), C-7 (160.6) and C-9 (156.2). A methoxyl group was assigned to C-7 based on HMBC correlations between a peak at 3.91 ppm in the ^1^H NMR spectrum and the ^13^C peak at 160.6. The protons at CH3-6 and H-8 also correlate with this peak at 160.6 supporting its assignment to position 7. As with **2**, peak characteristics of glucose were clearly observed in the carbon spectrum (61.1–99.3 ppm), indicating a glycoside. The glycoside is linked through the oxygen atom at position 5 due to the fact that the characteristic resonance of the 5-OH (∼13 ppm as observed in **1** and **2**) is not observed in the ^1^H NMR spectrum of **3**.

*δ* 1H (400 MHz, DMSO) 10.08 (1H, s, OH-4′), 9.06 (1H, s, OH-3), 8.07 (2H, d, *J* = 8.2 Hz), 7.11 (1H, s, H-8), 6.94 (2H, d, *J* = 8.5 Hz), 5.66 (1H, s), 5.43 (s), 5.15 (s), 5.10 (s), 4.82 (1H, d, *J* = 7.9 Hz), 4.76 (s), 3.91 (3 H, s, CH3-6), 3.78 (m), 3.48 (m), 3.46–3.20 obscured by H_2_O, 3.16 (m), 2.30 (s). *δ* 13C 176 (C-4), 160.6 (C-7), 159.0 (C-4′), 156.2 (C-9), 153.9 (C-5), 144 (C-2), 129.2 (C-2′/6′), 122.1 (C-1′), 115.6 (C-3′/5′), 107.5 (C-6), 104.2 (C-10), 99.6 (C-8), 99.3 (C-1″), 77.8, 76.1, 73.8, 70.3, 61.1, 56.2 (MeO-7) and 8.0 (Me-6).

### Oestrogenic and anti-oestrogenic activities of isolated compounds

**Figure 4.**
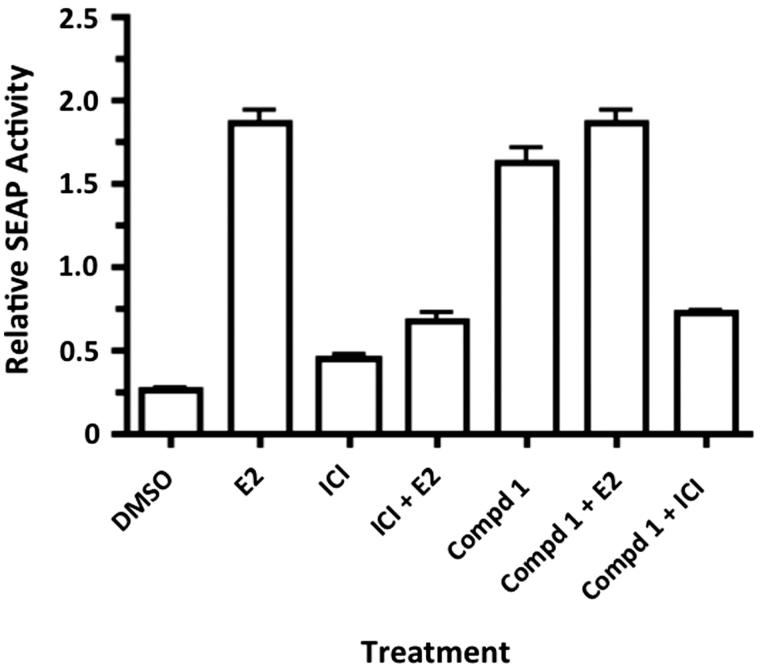
Compound **1** (1 μM) enhances expression of an oestrogen responsive reporter gene in transiently transfected MCF-7 cells (*p* < 0.05, *n* = 8). This effect was inhibited by the ER antagonist ICI 182,780 (1 μM) (*p* < 0.05), demonstrating that **1** acts through the ER. E2 was tested at (10 nM). Three independent experiments were performed, and all tests were performed in triplicate. Data were analysed using one-way ANOVA followed by Tukey’s multiple comparison test. DMSO vs. E2 *p* < 0.001; DMSO vs. **1***p* < 0.001; E2 vs. ICI + E2 *p* < 0.001; **1** vs. **1 **+ICI *p* < 0.001.

The oestrogenic activity of **1** was determined *in vitro* by competitive oestrogen receptor binding assay and functionalized cell-based, oestrogen-responsive reporter gene assay. Compound **1** demonstrated binding to ERβ with an IC_50_ of 0.39 mM. Treatment of transiently transfected MCF-7 cells with **1** at 1 μM significantly enhanced the expression of the oestrogen responsive SEAP reporter gene compared to the control (Figure 4). This enhanced expression was observed in the presence and absence of 10 nM estradiol (E2), and was attenuated by the ERβ antagonist ICI 182,780 (1 μM). The combination of **1** and E2 did not enhance SEAP activity, suggesting that the combination was not additive or synergistic.

**Figure 5.**
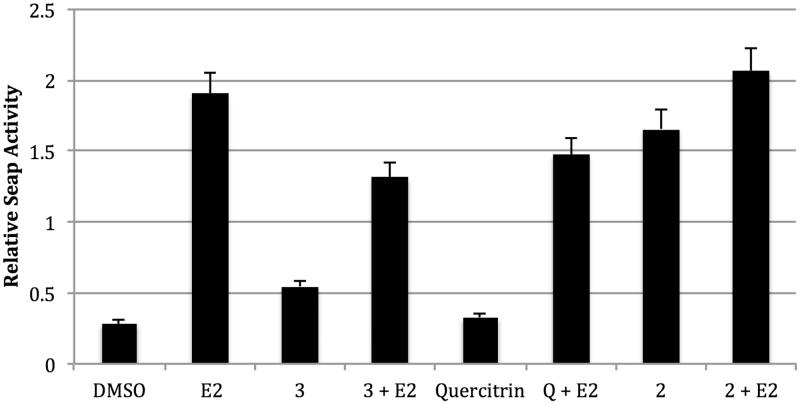
Treatment of transfected MCF-7 cells. MCF-7 cells were treated with *Pimenta* compounds at 1 μM for 24 h. Statistics were performed using one-way ANOVA followed by Tukey’s multiple comparison test. DMSO vs. E2 *p* < 0.001; E2 vs. 3 + E2 *p* < 0.001.

In the functionalized E2-dependent SEAP assay, neither quercitrin nor **3** alone significantly enhanced SEAP activity in MCF-7 cells. However, **2** significantly increased SEAP activity, suggesting oestrogen-agonist effects. Quercitrin and **3** showed some significant anti-oestrogenic effects in the SEAP reporter assay when combined with E2 (Figure 5).

### Cytotoxicity in MCF-7, AGS, NCI-N87 and SK-BR3 cells

The extract, fractions and pure compounds were tested in MCF-7 breast cancer cells, and gastric cancer cell lines AGS and NCI-N87. The IC_50_ of *Pimenta dioica* extracts and compounds is presented in Table 2. The extract and 50% methanol fraction were moderately active in MCF-7 and NCI-N87 but weakly active in AGS cells (Table 2). Of the four compounds tested, only **2** showed cytotoxic effects on the three cell lines (Table 2).

**Table 2.** IC_50_s of the *P. dioica* extract, 50% fraction, and four compounds against the cancer cell lines MCF-7, AGS and NCI-N87.

	IC_50_ (μg/mL)							
Cell line	PD MeOH	PDF-50%MeOH	Cmpd **2**	Cmpd **3**	Quercitrin	Cmpd **1**	5FU	Doxo
MCF-7	53.16	55.42	54.27	>100	>100	>100	1.37	
AGS	91.32	58.77	38.13	>100	>100	>100	18.64	0.079
NCI-N87	367.49	39.36	51.22	>100	>100	>100	0.097	0.048

**Figure 6.**
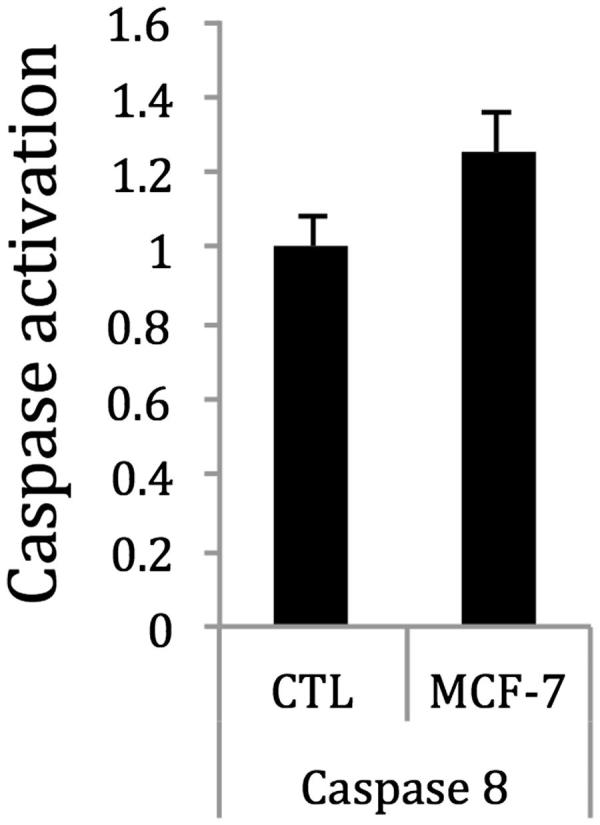
Treatment of MCF-7 cells with **2** at the IC_50_ concentration weakly enhanced the activity of caspase 8 after 2 h, as compared with control. Thus, **2** may induce apoptosis in MCF-7 breast cancer cells through the activation of caspase 8. Control cells were treated with vehicle solvent only.

The extract induced apoptosis in MCF-7 cells via caspase 8, but had no effect on caspase 3/7 in MCF-7 cells (data not shown). Treatment of MCF-7 cells with **2** inhibited cell proliferation and induced apoptosis, as well as caspase 8 activity (Figure 6).

### Quantitative polymerase chain reaction for apoptotic gene expression in MCF-7 cells

**Figure 7.**
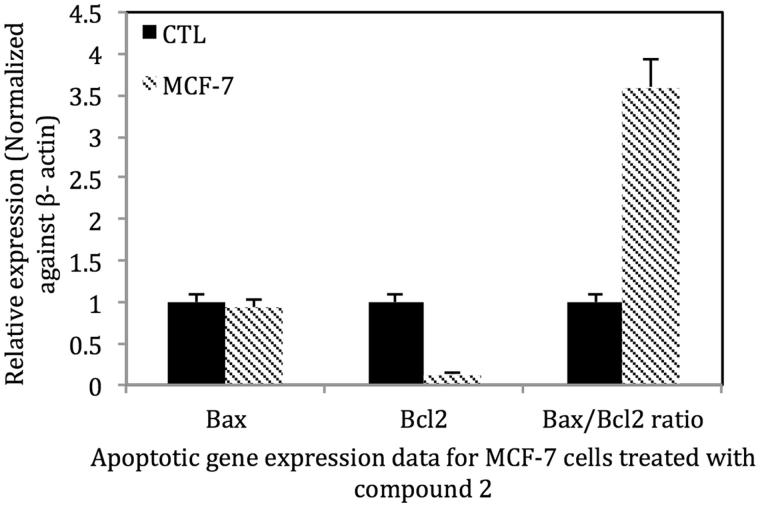
Treatment of MCF-7 (ER+) cells with **2** at the IC_50_ for 24 h decreased the expression of Bcl-2, the anti-apoptosis gene and increased the Bax/Bcl-2 ratio to ∼3.5 indicating that the extract induces apoptosis in MCF-7 cells via the intrinsic pathway. Control MCF-7 cells were treated with vehicle solvent only. For Bcl2 mRNA *p* < 0.001 MCF-7 vs. control; for the Bax/Bcl2 ratio *p* < 0.001 MCF-7 vs. control.

Treatment of MCF-7 cells with **2** at the IC_50_ for 24 h decreased the expression of Bcl-2 mRNA, but had little effects on Bax mRNA expression. The alteration in Bcl-2 expression significantly increased the Bax/Bcl-2 ratio favouring apoptosis (Figure 7).

### *Pimenta dioica* extract inhibits the growth of Sk-Br3 breast cancer cells

**Figure 8.**
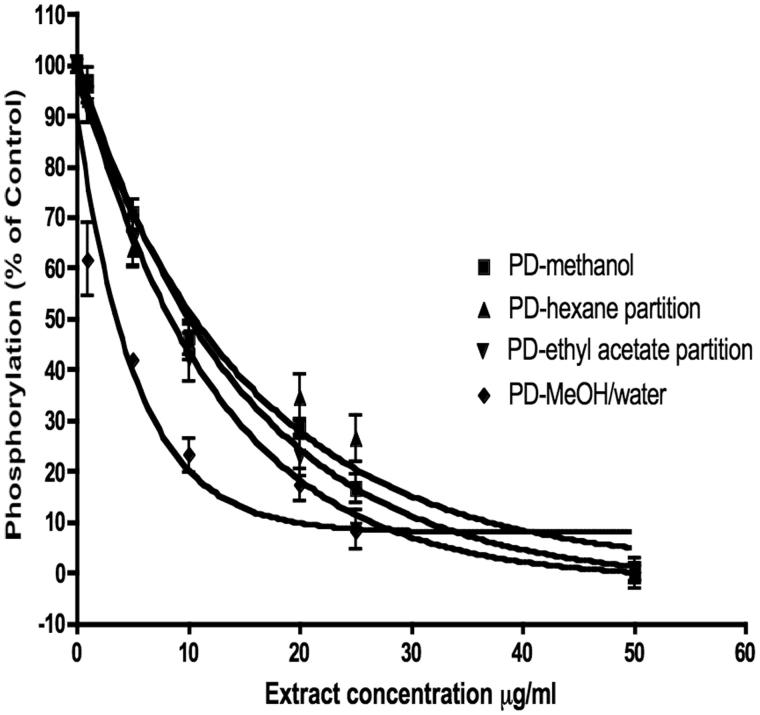
Inhibition of HER2/neu (erbB2) tyrosine kinase by *P. dioica* extracts and fractions. Phosphorylation of poly-GluTyr by purified erbB2 intracellular domains was measured by immunoassay with antiphosphotyrosine antibodies. The 50% methanol fraction was the most active with an IC_50_ of 5.5 μg/mL.

**Figure 9.**
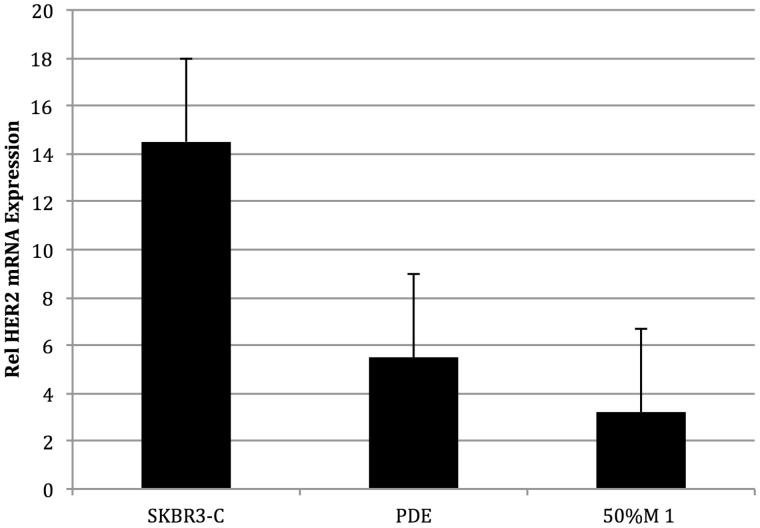
SKBr3 cells treated for 4 h with vehicle solvent, *P. dioica* extract (PDE), and 50% methanol fraction (50% M 1) (*n* = 9). Both PDE and the 50% M 1 inhibited the expression of HER2 mRNA by more than 50%.

The MeOH extract also inhibited SK-BR3 (ER-, high HER2) cell proliferation with an IC_50_ of 12.5 μg/mL. Using a high-throughput HER2 assay, the activity was determined to be in the 50% methanol/water fraction, which also inhibited the activity of HER2 tyrosine kinase (Figure 8). Since PD reduced HER2 tyrosine kinase activity, we also assessed the effects of the active fraction on HER2 mRNA levels. Treatment of SK-Br3 cells with the extract at 10 μg/mL for 12 h reduced HER2 mRNA expression by 55%, the 50% MeOH fraction at 5 μg/mL reduced HER2 expression by 80% (Figure 9). These data suggest that the extract and fraction inhibit the growth of Sk-Br3 cells by reducing the activity and expression of HER2. Compounds **2** and **3** were isolated from the 50% methanol fraction, but unfortunately we did not have sufficient quantities of these compounds to test in the HER2 assay.

## Discussion

Our previous investigations of Costa Rican plants used to treat women’s reproductive health, including menopause, focused primarily on the oestrogenic and progestagenic activities of these plant extracts (Doyle et al. 2007, 2009; Locklear et al. 2007, 2010, 2017). Results of this work demonstrated that extracts of *P. dioica* not only bound to the oestrogen receptors, but also induced the transcription of oestrogen responsive genes in MCF-7 cells (Doyle et al. 2009). However, while treatment of MCF-7 cells with *P. dioica* extract enhanced pS2 gene expression suggesting oestrogen agonist effects, in the presence of E2, the extract also reduced the expression of PR and PTGES mRNAs, suggesting possible oestrogen antagonist activities as well (Doyle et al. 2009). Since, the gene PTGES encodes for prostaglandin E2 synthase 1 (mPGES-1), a microsomal enzyme that is up-regulated in pre-malignant and malignant breast disease, our data suggested that the extract also might have activity against breast cancer (Doyle et al. 2009). In addition, allspice is also used in Costa Rica to treat dyspepsia, and experimental data show that *P. dioica* extracts inhibit the growth of *Helicobacter pylori in vitro* (Ali et al. 2005). Since the bacterium *H. pylori* has been designated a carcinogen by IARC, and is also well known to be associated with gastric, duodenal and colon cancers, we were further interested in assessing the effects of the *P. dioica* extract on gastric cancer cell proliferation *in vitro* (IARC 1994; Breuer-Katschinski et al. 1999; Scheiman and Cutler 1999; Shmuely et al. 2001; Correa 2002; Figueiredo et al. 2002).

In this work, we isolated and identified three new biologically active chromones including a 2-phenoxychromone, from the methanol extract of *P. dioica* leaves. This is the first report of a 2-phenoxychromone having been isolated from the family Myrtaceae. Compounds **1** and **2** significantly up-regulated the expression of an oestrogen responsive reporter gene at a concentration of 1 μM in transiently transfected MCF-7 cells (*p* < 0.05). For **1**, these effects were reduced by the addition of the ER antagonist ICI 182,780, suggesting that despite relatively weak binding to ERβ, **1** is acting through the ER, and thus is a partial ERβ agonist. Interestingly, other researchers investigated the biological activity of the essential oil of allspice, and found that one of its chemical constituents, eugenol, bound to oestrogen receptors, but did not induce expression of oestrogen-responsive genes in Ishikawa cells (Howes et al. 2002). The oestrogen agonist effects of *P. dioica* may explain why women in Costa Rica use this herb to manage menopausal symptoms, such as hot flashes and night sweats. However, neither **3** nor quercitrin increased the expression of the SEAP reporter gene in MCF-7 cells, but instead reduced reporter gene expression in the presence of E2, suggesting partial oestrogenic antagonist activity.

In female physiology, oestrogens play an essential role in a broad range of organs and tissues, including brain, breast, bone, central nervous system and the urogenital system (McDonnell and Norris 2002; Gardner et al. 2008). Oestrogens exert their effects through two nuclear subtypes, ERα and ERβ, both of which act as ligand-dependent transcriptional activators (McDonnell and Norris 2002). Activation of ER responsive genes occurs after direct binding of ER plus ligand to the ERE in gene promoters or by indirect binding through interactions with other transcriptional factors such as AP1 or NFκB (DeNardo et al. 2005). Changes in ER transcriptional target expression lead to downstream biological effects that include secondary effects that are induced by the biological activities of direct transcriptional targets. We chose to investigate the oestrogenic activity of **1** as mediated through ERβ rather than ERα because of the difference in the physiological response resulting from activation of each of these receptor subtypes. The proliferative effects of oestrogens in tissues such as the breast and uterus are mediated through the ERα subtype, while ERβ has an antiproliferative and pro-apoptotic effect in these tissues (Warner et al. 2017). In addition, the beneficial effects of oestrogen with regards to menopause, including a reduction of menopausal symptoms (hot flashes and night sweats) are mediated through ERβ (Tagliaferri et al. 2016; Warner et al. 2017). A recent phase II clinical trial, designed to test the efficacy of a selective ERβ agonist in reducing frequency of hot flashes in postmenopausal women, reported a median reduction in moderate to severe hot flashes of 71% of women (Tagliaferri et al. 2016). Other experimental studies showed that phytoestrogens enhance E2-induced transcription by increasing the expression of ERβ (Gardner et al. 2008; Mahmoud et al. 2015). Thus, a greater number of receptors may be activated when cells are treated with E2 in combination with a phytoestrogen, than with E2 alone. A proposed mechanism for the increase in ERβ expression is demethylation of the ERβ promoter through the mitigation of DNA methyltransferases, enzymes responsible for silencing of genes through hypermethylation of their promoter regions (Mahmoud et al. 2015).

In addition to up-regulating receptor expression, the ERα has a larger ligand-binding site than ERβ, and as a result some ligands that bind both receptor subtypes may activate ERα, but not ERβ (Pike et al. 1999; Polasek et al. 2013). Compound **1** shares many structural similarities with known ligands of both ER subtypes, specifically flat geometry and hydroxyl groups on the A and B rings separated by a distance of 9.7–12.3 Å (Polasek et al. 2013). It has been further suggested that flavonoids should be hydroxylated at positions 4′, 5 and 7 for optimal ER binding (Polasek et al. 2013). Compound **1** has hydroxyl groups in the optimal positions, but the distance between hydroxyl groups on the A and B rings is increased compared with flavonoids due to the ether linkage at C2 (Figure 1). However, the ether linkage also provides a hinge that may enable the 4′ hydroxyl group to orient itself in such a way that the interaction with the binding site is optimized. Although the oestrogenic effects of specific flavonoids and isoflavonoids, such as genistein, are well known, there has been little investigation of the oestrogenicity of naturally occurring members of the relatively rare 2-phenoxychromones. Polasek et al. (2013) isolated a 2-phenoxychromone from the leaves of *Peltophorum pterocarpum* (DC.) K.Heyne (Fabaceae) that bound both ER subtypes, but it did not induce transcription of oestrogen-responsive reporter genes through either receptor subtype. Researchers assessed the ER-binding and antiproliferative effects of several synthetic flavonoids containing an oxygen atom at carbon 2, which served as a ‘hinge’ to direct an ethoxy amine side chain to the ER binding site (Kim et al. 2003; Davis et al. 2008). Thus, there has been some interest in the oestrogenicity of compounds with structural characteristics similar to the 2-phenoxychromones, though structure–activity relationship studies have not been performed to determine the specific structural elements that are necessary for activation of ERα and ERβ by 2-phenoxychromones.

One of the serious issues associated with the ingestion of oestrogens and phytoestrogens is the possibility of enhancing the oestrogen-responsive cancer risk in women with familial history of the disease or with active breast or endometrial cancers. It is well known that oestrogens regulate the proliferation of ERα positive breast cancer cells through the regulation of many gene networks and signalling pathways (Saxena and Dwivedi 2012). To this end, we also investigated the effects of the *P. dioica* extract, fractions and compounds in two breast cancer cell lines MCF-7 and SK-BR3. The extract and a 50% MeOH fraction inhibited the growth of both MCF-7 and SK-BR-3 breast cancer cells, but was less effective in MCF-7 cells that express only basal levels of HER2. However, since **2** induced apoptosis in MCF-7 cells, we also investigated some of the well known signalling factors involved in apoptosis, including the Bcl-2 and caspase family of proteins (Kugu et al. 1998). The Bcl-2 proteins are located on the nuclear, outer mitochondrial and endocytoplasmic reticulum membranes and are key regulators of apoptosis involving the mitochondrial membrane (Kugu et al. 1998; Liu et al. 2010). The Bcl-2 family of proteins includes the anti-apoptotic proteins Bcl-2 and Bcl-x. Reductions in the expression of Bcl-2 or increases in Bax expression induce apoptosis (Liu et al. 2010). In addition, the ratio of Bcl-2 to Bax in the dimer impacts the apoptotic status. If the ratio of Bcl-2 > Bax, the dimer prefers cell survival; if the ratio of Bcl-2 < Bax, apoptosis is dominant. In this work, we demonstrate that treatment of MCF-7 cells with **2** enhanced both caspase 8 activity and decreased the expression of Bcl-2 mRNA, but had little effects on Bax mRNA expression. The reduction in Bcl-2 mRNA expression significantly increased the Bax/Bcl-2 ratio to be more favourable towards apoptosis in MCF-7 cells.

In addition to MCF-7 cells, the extract and 50% aqueous methanol fraction inhibited the growth of ER negative SK-BR3 cells. ERα negative breast cancer cells proliferate independently of oestrogen signalling through other signalling pathways such as the EGFR, and downstream PI3K/Akt/mTOR, NF-κB signalling pathways (Saxena and Dwivedi 2012). The Human Epidermal Growth Factor Receptor (HER) family is made up of four trans-membrane receptor tyrosine kinases that impact intracellular signalling pathways regulating cell proliferation and differentiation (Saxena and Dwivedi 2012). The second member, HER2, is of importance in breast cancer as overexpression or gene amplification of the HER2 oncogene is closely associated with aggressive tumour progression and poor prognosis in breast and ovarian cancer (Baselga and Cortes 2005; Baselga and Swain 2009). HER2 overexpression is found in both the primary tumour and in metastatic sites, indicating that anti-HER2 therapy may be effective at all disease sites. In preliminary experiments, we showed that the 50% MeOH fraction inhibited the activity of the HER2 tyrosine kinase, and reduced the expression of the HER2 mRNA in SK-BR3 cells. Thus, it is possible that *P. dioica* acts as a HER2 inhibitor, but further research is needed to identify the active constituents.

Finally, in gastric cancer cells, the extract, the 50% MeOH fraction and **2** inhibited AGS and NCI-N87 cell proliferation, suggesting potential effects against gastric cancer. Other researchers reported that polyphenols isolated from a *P. dioica* leaf methanol extract inhibited the proliferation of Hep-G2 liver cancer and HCT-116 colon cells, but had a lesser effect on MCF-7 cells (Zhang and Lockeschwar 2012). Our data support these previous observations, as the extract was more effective in gastric cancer cells than MCF-7 cells. Since *P. dioica* is used in Costa Rica and other countries to manage gastritis, dyspepsia and other gastrointestinal disorders (Zhang and Lockeschwar 2012), and *P. dioica* extracts and **2** inhibit the growth of *H. pylori*, it is also possible that allspice/pure compounds may be developed for gastric cancers.

## Conclusions

Allspice is used in Costa Rican ethnomedicine for the management of women’s reproductive health issues, menopause, gastritis and a number of other conditions. Literature on the safety and efficacy of this plant is limited. Isolation and identification of new compounds from the leaves of *P. dioica* with oestrogenic effects corroborates the Costa Rican traditional use in the treatment of menopausal symptoms. The inhibitory impact of the extract, fractions and pure compounds on breast and gastric cancer cells suggests that this plant has potential to be developed for the prevention or treatment of these cancers, and further suggests that this plant may not increase the risk of breast cancer in women using it to treat menopause.
